# Calcium-Sensing Receptor Mediates β-Amyloid-Induced Synaptic Formation Impairment and Cognitive Deficits *via* Regulation of Cytosolic Phospholipase A2/Prostaglandin E2 Metabolic Pathway

**DOI:** 10.3389/fnagi.2020.00144

**Published:** 2020-06-24

**Authors:** Chenxi Feng, Xiaoming Bao, Ling Shan, Yunxiang Ling, Yanfei Ding, Jia Wang, Yanzi Cao, Qinwen Wang, Wei Cui, Shujun Xu

**Affiliations:** ^1^School of Medicine, Ningbo University, Zhejiang Provincial Key Laboratory of Pathophysiology, Ningbo, China; ^2^Children’s Hospital of Soochow University, Suzhou, China; ^3^HwaMei Hospital, University of Chinese Academy of Sciences, Ningbo, China; ^4^Ningbo Institute of Life and Health Industry, University of Chinese Academy of Sciences, Ningbo, China; ^5^Netherlands Institute for Neuroscience, Royal Netherlands Academy of Arts and Sciences, Amsterdam, Netherlands

**Keywords:** Alzheimer’s disease (AD), β-amyloid peptide (Aβ), calcium-sensing receptor (CaSR), synapse formation, cytosolic phospholipase A2, prostaglandin E2

## Abstract

Calcium-sensing receptor (CaSR) is a G protein-coupled receptor (GPCRs). Soluble β-amyloid peptide (Aβ) is one of the orthosteric modulators of CaSR, while, the role and underlying mechanism of CaSR in cognitive decline in Alzheimer’s disease (AD) is unclear. In this study, molecular technology such as live-cell imaging combined with behavioral tests were used to explore the role and the underlying mechanism of CaSR in the cognitive deficits in AD mice. The expression levels of CaSR were increased both in AD mice and Aβ_1–42_ (β-amyloid protein)-treated primary cultured neurons. Pharmacological inhibition of CaSR ameliorated recognitive and spatial memory deficits of Aβ_1–42_-oligomer-treated mice in a dose-dependent manner. Pharmacological inhibition of CaSR or down-regulation of the expression of CaSR by CaSR-shRNA-lentivirus prevented the impairment of filopodia, and the synapse induced by oligomeric Aβ_1–42_. The contents of cytosolic phospholipase A2 (cPLA2) and prostaglandin E2 (PGE2) in hippocampal neurons and tissue were increased after treatment with Aβ_1–42_ oligomers. Inhibition or down-regulation of CaSR mediates Aβ-induced synapse formation and cognitive deficits partially, through the activation of the cPLA2/PGE2 pathway. This study provides novel insights on CaSR, which is a promising therapeutic target for AD.

## Introduction

Alzheimer’s disease (AD) is one of the most common neurodegenerative diseases with a slow and gradual deterioration of the memory that affects language, personality, and cognitive control (Wyss-Coray and Rogers, 2012; Mhatre et al., 2014; Dos Santos Picanco et al., 2018). AD is a severe threat for human health, along with an aging population, however, the pathogenesis of it is still unclear. Mounting evidence shows that the accumulation of β-amyloid peptide (Aβ) is an important contributing factor in the pathology of AD (Baek et al., 2017; Choi et al., 2017). Aβ has different aggregated forms, including monomers, oligomers, protofibrils, and mature fibrils (Ahmed et al., 2010). It is well demonstrated that soluble oligomers of Aβ is the pertinent toxic form of Aβ (Wang H. C. et al., 2016; Wang T. et al., 2016). Both others, and our previous studies have shown that soluble Aβ oligomers could decrease the number of dendritic spines and inhibit the long-term potentiation (LTP), leading to the decline of cognitive function in AD mice (Price et al., 2014; Jiang et al., 2016). Disrupting synapse function plays an important role in the memory deficits of AD, which stands out in early AD pathological changes (Price et al., 2014; Teich et al., 2015; Wang X. et al., 2018). Therefore, it is a promising strategy to consider amelioration of synaptic impairment induced by Aβ oligomers as the target of prevention or treatment of AD.

Aβ is also one of the allosteric agonists of calcium-sensing receptor (CaSR). Aβ has been shown to interact directly with CaSR *via* a proximity ligation assay (Diez-Fraile et al., 2013; Leach et al., 2015). CaSR, a member of the G protein-coupled receptor (GPCRs) C family, is a seven-transmembrane protein (Brauner-Osborne et al., 2007; Conigrave and Ward, 2013; Summers, 2016). CaSR has been detected in the hippocampus which is an important brain structure that is essential for spatial memory, language learning, and episodic memory (Ferry et al., 2000). CaSR is mainly localized in nerve endings in the neurons and is involved in regulating brain excitability (Chen et al., 2010). CaSR activation depends on the persistent interaction with its agonists. At physiological conditions, CaSR is partially activated (Ruat and Traiffort, 2013; Díaz-Soto et al., 2016). In cultured cells, Aβ-activated CaSR could cause excessive release of Aβ (Armato et al., 2013; Dal Prà et al., 2014). The expression of CaSR was significantly increased in AD transgenic mice (Leach et al., 2015; Gardenal et al., 2017). The role of CaSR in AD is unclear and the cellular mechanisms have not been well characterized. As a promising therapeutic target, we therefore evaluated the role of CaSR in cognitive deficits in the mouse model of AD and its underlying cellular mechanisms. The effects of CaSR on oligomeric Aβ-induced synaptic injury are unknown. In the current study, we have also evaluated the role and the underlying mechanisms of CaSR in Aβ-mediated synaptic impairment.

## Materials and Methods

### Aβ1–42 Oligomers Preparation

Preparation of soluble Aβ_1–42_ oligomers was done according to the protocol previously described (Jiang et al., 2016; Ding et al., 2019). One milligram of Aβ_1–42_ (Bachem, Cat# H-1368.1000) powder was dissolved in 400 μl ice-cold 1,1,1,3,3,3-hexafluoro-2-propanol (HFIP; Aladdin, Cat# K1625063), and incubated at room temperature for 20 min. Hundred microliter of this complete solution was diluted into 900 μl of deionized water to a final concentration of 0.25 g/l. After centrifugation at 14,000 *g* for 15 min, the supernatant was collected and the HFIP was completely evaporated. Then the collected supernatant was kept stirring for 48 h at room temperature. A 50 μM Aβ_1–42_ solution was obtained and stored at 4°C. The preparation is the combination of low molecular weight forms of soluble Aβ (Chunhui et al., 2018).

### Animals

ICR mice (RRID:IMSR_CRL:22) or B6C3-Tg (APPswe/PSEN1dE9) mice were used in our experiments. Breeding pairs of APPswe/PSEN1dE9 transgenic mice were originally purchased from Jackson Laboratories, USA. A breeding colony of APPswe/PSEN1dE9 mice was established at the Medical School of Ningbo University. All experimental animals were housed in a temperature and humidity-controlled animal facility (22 ± 3°C, 60% ± 5%) with a 12 h light and dark cycle and free access to standard chow and water. Experiments were carried out in accordance with the National Institute of Health Guide for the Care and Use of Laboratory Animals (NIH Publications No. 80-23, revised 1996) and approved by the Institutional Animal Care and Use Committee of the Ningbo University. The approval number for the animal experiments is SYXK (ZHE) 2013-0191. Genotypes of APPswe/PSEN1dE9 mice were analyzed as follows: DNA was isolated from the tail tip of each mouse and PCR was performed using the following primer pairs: APP, forward primer5′-GACTGCCACTCGACCAGGTTCTG-3′, reverse primer 5′-CTTGTAAGTTGGATTCTCATATCCG-3′; PS1, forward primer 5′-GTGGATAACCCCTCCCCCAGCCTAGACC-3′, reverse primer 5′-AATAGAGAACGGCAGGAGCA-3′. APPswe/PSEN1dE9 transgenic mice and wild type mice were identified by agarose gel electrophoresis. In each identification experiment, both positive and negative controls were designed.

### Animal Surgery

Two-month-old healthy male ICR mice (25–30 g) were pseudo-randomly assigned to the experimental groups using a random number generated from Excel, and all mice were marked by staining in different parts of the back. A pre-test open field experiment was first performed on all mice to determine the locomotor activity before the formal experiments and to exclude those with an obvious movement disorder. No obvious movement disorder was found among the mice. Thus, no mice were excluded. ICR mice were anesthetized by intraperitoneal administration of sodium pentobarbital (50 mg/kg) before they were placed in a stereotaxic apparatus (Stoelting, Wood Dale, IL, USA). Cannulas (RWD Life Science, Shenzhen, China) were surgically implanted into bilateral hippocampal regions of the mice using the following coordinates: AP −1.7 mm from Bregma; ML ± 1.0 mm from the midline; DV −1.5 mm from pia mater. After 7 days of post-operative recovery, the minipump needle tips were inserted into the ventricle through cannulas to inject pharmaceuticals. Experimental mice were given three consecutive infusions of Aβ_1–42_ (4 μmol/kg) and/or NPS 2143 (Sigma, Cat# SML0360, 0.08 or 0.16 μmol/kg), while the control mice received saline injections instead of the drugs.

### Behavior Tests

The novel object recognition (NOR) task, consisting of a familiarization phase and a test phase, was carried out in an open-field arena (60 × 60 × 15 cm) on the 11th to 12th day after the first injection. On the first day, they were familiarized with two identical objects for 5 min. On the second day, one of the objects was replaced by a novel one with a different shape and color, and the mice were allowed to explore the arena for 5 min. To ensure the absence of olfactory cues, the open-field arena and the objects were cleaned thoroughly. Exploration was defined as sniffing or touching the objects. The distance between the nose and object was no more than 2 cm. If the mice traveled around or sat on the objects, this was not defined as object recognition. The discrimination index, the ratio of the amount of time spent exploring any one of the two objects (training session) or the novel object (retention session), over the total time spent exploring both objects, was used to measure the cognitive function of animals.

A Morris water maze (MWM) was performed as described (Jiang et al., 2016). Briefly, the equipment included a pool with a diameter of 110 cm that was filled with opaque water at approximately 22 ± 1°C. Spatial memory was assessed by recording the latency time for the animal to escape from the water onto an escape platform during the place navigation phase. At the learning phase, mice were given 90 s to find the hidden platform that was 1 cm below the water surface. The place navigation test of the MWM, which consisted of four trials (interval 20–30 min) each day, took place during the 14th day to the 17th day and the latency time was recorded. On the 18th day, the platform was removed from the maze. A probe trial was conducted to measure the trajectories and entries of mice to the target quadrant with a video tracking system (Ethovision XT). The assessor was blinded to the experimental conditions for analysis of the behavioral tests.

### Primary Hippocampal Neuronal Cultures

Neurons were derived from the dissociating hippocampus of newborn ICR mice (RRID:IMSR_CRL:22). To achieve dissociated and single cells, the tissue was cut into tiny pieces, and the cells within released by a mild treatment with 0.125% (v/v) trypsin (Gibco, Cat# 11668–019) for 15 min at 37°C. Isolated neurons were then planted on coverslips (Fisherbrand, Cat# 12–545–83), pre-coated with poly-D-lysine (Sigma, Cat# P0899–10MG) in Dulbecco’s modified Eagle media (Gibco, Cat# 11995065) containing 10% (v/v) FBS (Gibco, Cat# 10099141), 10% (v/v) F-12 (Gibco, Cat# 11765054), and 1% (v/v) of penicillin-streptomycin solution (Solarbio, Cat# P1400). After the cells were incubated at 37°C, 5% (v/v) CO_2_ for 24 h, the medium was changed to Neurobasal medium supplemented with 2% (v/v) B27 (Gibco, Cat# 17504044) and 1% (v/v) L-glutamine (Gibco, Cat# 25030–081). The medium was half-replaced every 3 days. At the 5th day *in vitro* (DIV 5), cytosine arabinofuranoside (Sigma, Cat# 147–94–4) was added at a final concentration of 2 μM to decrease glial cell growth. The neurons were transfected with farnesylated enhanced green fluorescent protein (F-GFP) and GFP-actin using Lipofectamine 2000 (Invitrogen, Cat# 11668–019). At DIV 7 or DIV 14 of culture, NPS 2143 was added to cultures of hippocampal neurons at a final concentration of 0.1 μM for 2 h. After the treatment of NPS 2143, neurons were incubated with Aβ_1–42_ (final concentration 0.5 μM) for 3 h. For the effect of Aβ_1–42_ on the expression of CaSR, primary hippocampal neurons (DIV 7) were incubated with Aβ_1–42_ for 36 h.

### Immunohistochemistry and Immunocytochemistry

Mice were decapitated and their brains were removed after perfusion. After fixation with 4% paraformaldehyde (Solarbio, Cat# P1110), dehydration with 30% sucrose solution and after being embedded with an optimum cutting temperature compound (Solarbio, Cat# 4583), the brains were cut into 30 μm frozen brain slices. Cultured cells were also fixed with 4% paraformaldehyde at room temperature. After being washed with phosphate-buffered saline (PBS) three times, the brain slices or cultured cells were blocked with sheep serum at room temperature for 1 h, to saturate unspecific binding sites, and then permeabilized in PBS containing 0.01% (V/V) triton (Solarbio, Cat# T8200). The sections or coverslips were incubated overnight (4°C) with anti-postsynaptic density 95 antibody (PSD 95, 1:200, Abcam, Cat# ab2723), anti-synaptotagmin-1 antibody (1:200, Millipore, Cat# AB5600–50UL), anti-MAP2 antibody (1:200, CST, Cat# 8707), anti-CaSR antibody (1:200, Santa Cruz Biotechnology, Cat# sc-47741), or DAPI (1:5000, Beyotime, Cat# C1002). After thorough washing, sections or coverslips were incubated with Alexa-488 anti-mouse secondary antibodies (1:1,000, Invitrogen, Cat# A-10680) or Cyanine 5 anti-mouse secondary antibody (1:1,000, Invitrogen, Cat# A10524) and Alexa-546 anti-rabbit secondary antibodies (1:1,000, Invitrogen, Cat# A11010) for 1 h at room temperature. Images of distal neuronal dendrites were captured by a confocal microscope (Olympus, Tokyo, Japan).

### Lentivirus Construction

The Lentivirus was purchased from Shanghai Genechem Company, Limited (China). The company used the following procedures: The coding sequence of the CaSR was from GenBank: NM_013803.3. For small hairpin RNA (shRNA) against mouse CaSR, vectors were constructed from the original plasmid GV lentiviral vector, and the GV118 serotype was selected; the reaction element sequence of which is U6-MCS-Ubi-EGFP. The target gene was inserted into the MCS element by HpaI and XhoI, two restriction endonucleases. The optimal target sequence (TCTTCATCAAGTTCCGAAA) was selected for small hairpin RNA (shRNA) against mouse CaSR, and a scrambled shRNA (TTCTCCGAACGTGTCACGT) served as a control. For viral packaging, the respective recombinant plasmids were cotransfected into 293T cells (ATCC, RRID:CVCL_0063). The GV stocks were titered by quantitative PCR, stored at −80°C of a titer of 10^9^ particles/ml and shipped with 20 μl in every tube. We then used a total of 5*10^5^ TU/ml of the virus to transfect the hippocampal neurons. All procedures were performed under a biosafety cabinet in a biosafety level 2 facility.

### Confocal Imaging and Analysis

At DIV 7 or DIV 14 of culture, living neurons were captured by a Fluoview-1000 confocal microscope. After drug treatments, the neurons were maintained in a recording chamber with extracellular solution (148.00 mM NaCl, 3.00 mM KCl, 3.00 mM CaCl_2_, 10.00 mM HEPES, and 8.00 mM glucose, pH 7.3) at room temperature. Digital images of GFP were collected on a Fluoview-1000 confocal microscope (Olympus) using a 60× oil objective lens without optical zoom at an excitation wavelength of 488 nm. They were analyzed using Fluoview-1000 software. All lengths of the secondary dendritic branches were measured by tracing their extension, and the filopodia and spines were counted. For all analyses, images were analyzed blind to treatments and data were collected from at least three independent experiments.

### Cytosolic Phospholipase A2 (cPLA2) and Prostaglandin E2 (PGE2) Assay

Cytosolic phospholipase A2 (cPLA2) or prostaglandin E2 (PGE2) levels in cultured hippocampal neurons and tissues were assayed using a commercial mouse cPLA2 (Qiaodu-Bio, Cat# CK-E92479M) or PGE2 ELISA Kit (Qiaodu-Bio, Cat# CK-E90213M), respectively. The hippocampal neurons and tissues were homogenized in ice-cold 70 μl RIPA (Solarbio, Cat# R0010) buffer. The samples were centrifuged at 13,000 *g* (4°C for 10 min). Ten micro-liter of the supernatant from the hippocampal homogenate was used to assay cPLA2 or PGE2 level, according to the manufacturer’s protocols. The content of cPLA2 was based on measures of absorbance at 450 nm/well in a 96 well plate reader (Thermo Scientific, USA).

### Western Blot Assay

The hippocampus was collected after the behavioral assessments. Tissues were homogenized in RIPA lysis buffer (Beyotime, Cat# P0013C) containing a Protease Inhibitor Cocktail (one tablet for every 50 ml RIPA lysis buffer, Roche, Cat# 11697498001) and a Phosphoprotease Inhibitor Cocktail (one tablet for every 50 ml RIPA lysis buffer, Roche, Cat# 4906845001), then, crushed by ultrasound for 10 min by an ultrasonic cell disruptor (Banoelin, Germany). After 30 min of being adequately crushed, the lysates were centrifuged at 12,000 rpm, for 30 min at 4°C and the supernatant fraction was used for the Western blot assay. The protein concentration in the supernatant fraction was determined using the BCA protein assay kit (Beyotime, Cat# P0012). Equal amounts of soluble protein (25 μg) were separated by 10% SDS-PAGE (Beyotime, Cat# P0456) and transferred onto poly-vinylidene fluoride (PVDF) membranes (0.45 μm, Millipore, Cat# IPVH08100). After blocking with 5% fat-free milk; Beyotime, Cat# P0216) for 1 h, membranes were incubated with rabbit anti-PSD 95 (1:1,000, Cell Signaling, Cat# 3409), mouse anti-synaptotagmine-1 (1:1,000, Abcam, Cat# 13259), and rabbit anti-β-actin (1:1,000, Cell Signaling, Cat# 4970) at 4°C overnight. Membranes were then incubated with HRP linked anti-rabbit Antibody (1:5,000, Cell Signaling, Cat# 7074) or HRP linked anti-mouse antibody (1:5,000, Cell Signaling, Cat# 7076), respectively. Target bands were detected and quantified with BeyoECL Plus (1:1, Beyotime, Cat# P0018) by Amersham imager 600 (GE Healthcare Life Sciences, USA).

### Quantitative RT-PCR

Total RNA was extracted from primary cultured hippocampal neurons and reverse transcribed into cDNA following standard experimental procedures. The relative transcript level of CaSR was measured by quantitative PCR using LightCycle 480VR II PCR (Roche, Switzerland) with specific primers (CaSR, forward primer 5′-TTGCAAGGGCCAATGGTGG-3′, reverse primer 5′-GCTTCCTGGGAAGACCCAT-3′; mouse-actin, forward primer 5′-AACAGTCCGCCTAGAAGCAC-3′, reverse primer 5′-CGTTGACATCCGTAAAGACC-3′).

### Statistical Analyses

Statistical analyses were performed with GraphPad PRISM software (GraphPad Software Inc., La Jolla, CA, USA, RRID:SCR_00298). Data were presented as mean ± SEM. Two-group comparisons were analyzed by a two-tailed student’s *t*-test. Multiple group data were analyzed using a one-way analysis of variance (ANOVA) followed by a Tukey *post hoc* test, with the exception of the data of the place navigation test of the MWM tests, which were analyzed by two-way repeated-measures ANOVA with Tukey *post hoc* comparisons. *P* < 0.05 was considered statistically significant. A test for outliers was not performed on the data.

## Results

### The Expression Levels of CaSR Are Increased in AD Mice and Aβ_1–42_-Treated Primary Hippocampal Neurons

**Figure 1:**
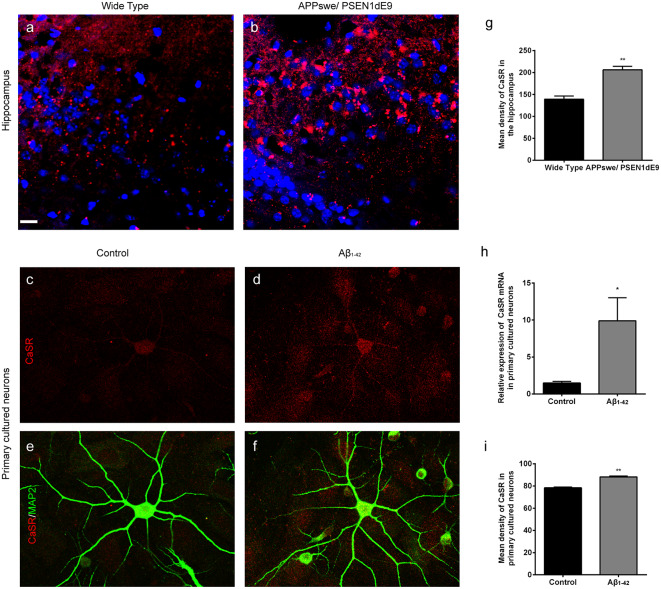
The expression levels of calcium-sensing receptor (CaSR) were increased in Alzheimer’s disease (AD) mice and β-amyloid peptide (Aβ_1–42_)-treated primary hippocampal neurons. **(A,B)** Immunostaining of CaSR (red) and DAPI (blue) in the hippocampus of wild type and APPswe/PSEN1dE9 transgenic mice. Scale bar 20 μm. **(C–F)** Immunostaining of CaSR (red) and neuronal marker MAP2 (green) in primary cultured neurons in the control and 0.5 μM Aβ_1–42_-treated group. **(G)** Quantitative comparison of the mean density of CaSR in the hippocampus of wild type and APPswe/PSEN1dE9 transgenic mice. **P < 0.01 vs. wild type group. Values represent mean ± SEM, *n* = 6 animals for each group. **(H)** Quantitative comparison of the mRNA levels of CaSR in control and Aβ_1–42_-treated neurons. *P < 0.05 vs. control group. *n* = 9 repeats from three independent primary cell culture preparations. **(I)** Quantitative comparison of the mean density of CaSR in control and Aβ_1–42_-treated neurons. **P < 0.01 vs. control group, *n* = 48–51 neurons from three independent primary cell culture preparations.

The expression levels of CaSR in APPswe/PSEN1dE9 transgenic mice and wild type mice were detected (Figures 1A,B). The expression levels of CaSR were significantly increased in the 9-month-old AD mice (*P* < 0.01, Figure 1G). The expression levels of CaSR in the control and Aβ_1–42_-treated primary hippocampal neurons were also detected (Figures 1C–F). Both the mRNA and protein levels were increased by Aβ_1–42_ treatment (*P* < 0.05, Figure 1H; *P* < 0.01, Figure 1I). The results demonstrated that Aβ_1–42_ treatment upregulates the expression level of CaSR.

### Pharmacological Inhibition of CaSR Prevents Dendritic Filopodium Loss Caused by Oligomeric Aβ_1–42_ in Hippocampal Neurons

**Figure 2:**
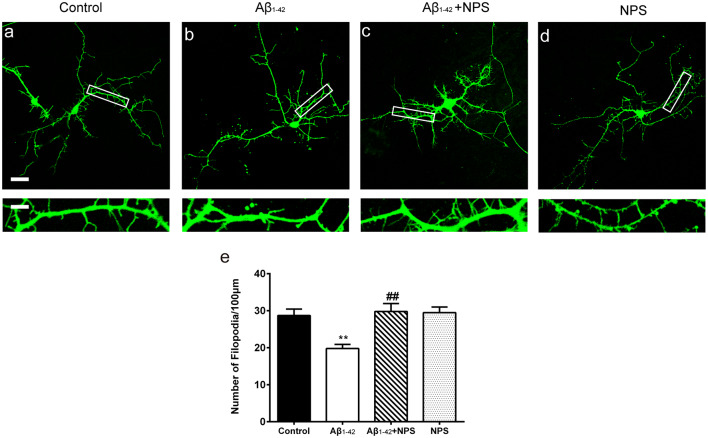
Pharmacological inhibition of CaSR protected hippocampal neurons from soluble Aβ_1–42_ oligomer-induced filopodium loss at day *in vitro* (DIV) 7. Structural morphology of filopodium of hippocampal neurons was displayed by co-transfection of F-GFP and GFP-actin. Fluorescent figures were displayed with the control group **(A)**, the 0.5 μM Aβ_1–42_ group **(B)**, the 0.5 μM Aβ_1–42_ + 0.1 μM NPS 2143 group **(C)** and the 0.1 μM NPS 2143 group **(D)**. Scale bar 20 μm and 5 μm. **(E)** Quantitative comparison of the density of dendritic filopodia of the four groups. **P < 0.01 vs. control group, ^##^*P* < 0.01 vs. Aβ_1–42_ oligomer-treated group. Values represent mean ± SEM, *n* = 21–25 neurons from three independent primary cell culture preparations.

To find out whether CaSR mediates Aβ_1–42_-induced early synapse formation impairment, we measured the density of dendritic filopodium of hippocampal neurons at DIV 7 treated with Aβ_1–42_ oligomers, in the presence or the absence of CaSR antagonist NPS 2143 the density of dendrite filopodium of the Aβ_1–42_-treated group was significantly reduced compared to the control group (*P* < 0.01, Figure 2). Treatment with NPS 2143 (0.1 μM) significantly ameliorated the reduction of filopodium density induced by Aβ_1–42_ oligomers (*P* < 0.01), while, NPS 2143 treatment alone did not alter the density of dendrite filopodium (*P* > 0.05, Figure 2).

### NPS 2143 Prevents Spine Loss and Synaptic Impairment Caused by Oligomeric Aβ_1–42_ in Hippocampal Neurons

**Figure 3:**
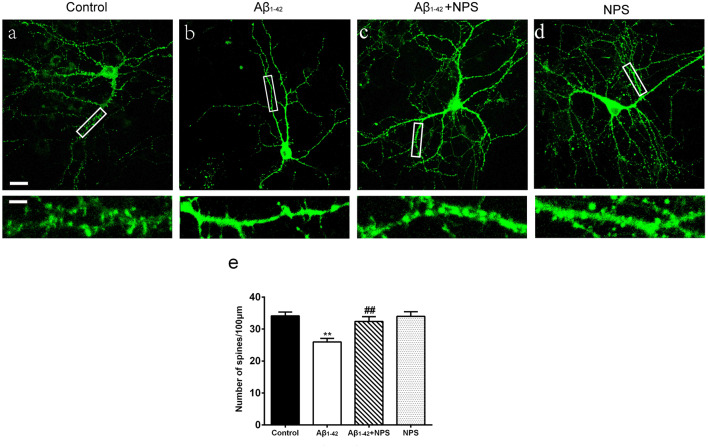
NPS 2143 protected hippocampal neurons from soluble Aβ_1–42_ oligomer-induced spine loss at DIV 14. Structural morphology of the spine of hippocampal neurons was displayed by co-transfection of F-GFP and GFP-actin. Fluorescent figures captured by confocal were displayed with the control group **(A)**, the 0.5 μM Aβ_1–42_ group **(B)**, the 0.5 μM Aβ_1–42_ + 0.1 μM NPS 2143 group **(C)** and the 0.1 μM NPS 2143 group **(D)**. Scale bar 20 μm and 5 μm. **(E)** Quantitative comparison of the density of the dendritic spine of the four groups. **P < 0.01 vs. control group, ^##^*P* < 0.01 vs. Aβ_1–42_ oligomer-treated group. Values represent mean ± SEM, *n* = 21–25 neurons from three independent primary cell culture preparations.

To further analyze the role of CaSR in synapse formation, the dendritic spine densities at DIV 14 were quantified in the control group, oligomeric Aβ_1–42_-treated group, oligomeric Aβ_1–42_ + NPS 2143 group and the NPS 2143 alone group. The oligomeric Aβ_1–42_-treated group prominently decreased the spine density (*P* < 0.01, Figure 3). Treatment with NPS 2143 (0.1 μM) significantly prevented the decreased spine density induced by Aβ_1–42_ oligomers (*P* < 0.01, Figure 3), while, NPS 2143 treatment alone did not alter the density of the spine (*P* > 0.05, Figure 3). These results suggest that CaSR is involved in dendritic spine loss caused by Aβ_1–42_ oligomers in hippocampal neurons.

**Figure 4:**
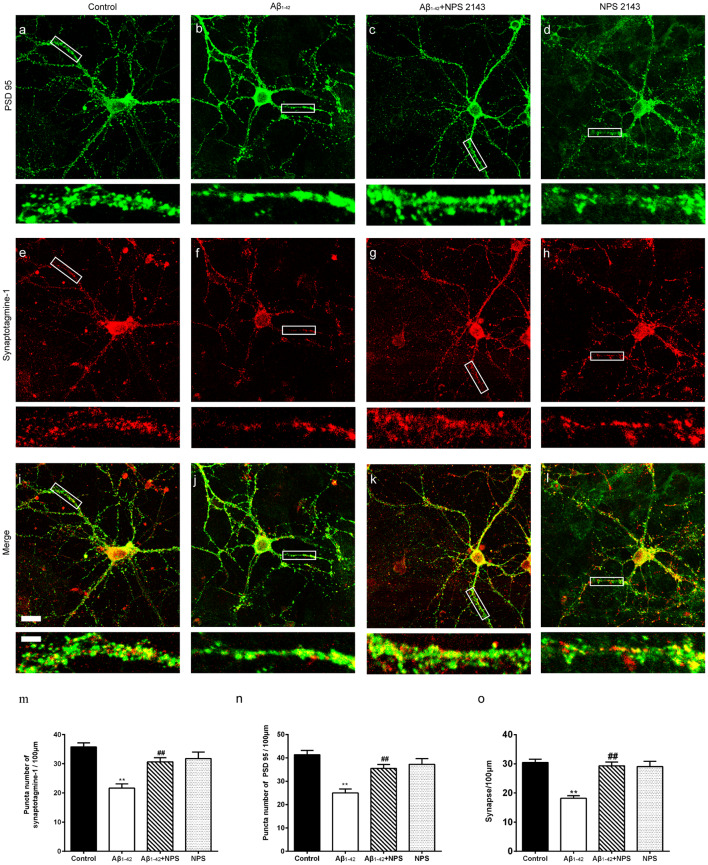
NPS 2143 prevented hippocampal neurons from soluble Aβ_1–42_ oligomer-induced synapse loss at DIV 14. Synapse was labeled through the co-localization of presynaptic marker synaptotagmine-1(red) and postsynaptic marker PSD 95 (green). Sample figures of the control group **(A,E,I)**, the 0.5 μM Aβ_1–42_ group **(B,F,J)**, the 0.5 μM Aβ_1–42_ + 0.1 μM NPS 2143 group **(C,G,K)**, and the 0.1 μM NPS 2143 group **(D,H,L)**. Scale bar 20 μm and 5 μm. **(M)** Quantitative comparison of the numbers of synaptotagmine-1-positive puncta of the four groups. **(N)** Quantitative comparison of the numbers of PSD 95-positive puncta of different groups. **(O)** Quantitative comparison of the density of dendritic synapse of the four groups. **P < 0.01 vs. control group, ^##^*P* < 0.01 vs. Aβ_1–42_ oligomer-treated group. Values represent mean ± SEM, *n* = 24–34 neurons from three independent primary cell culture preparations.

To confirm the effects of CaSR on synaptic impairment induced by Aβ_1–42_, the synapse density was captured by immunocytochemistry (Figures 4A–L). At DIV 14, anti-presynaptic marker synaptotagmine-1 and anti-postsynaptic marker PSD 95 specific antibodies were used, and puncta per 100 μm dendrite from secondary dendritic branches were analyzed. Compared with the control group, the numbers of synaptotagmine-1-positive puncta and PSD 95-positive puncta were significantly decreased after treatment with Aβ_1–42_ oligomers (*P* < 0.01, Figures 4M,N). The NPS 2143 (0.1 μM) treatment significantly prevented the decreased puncta numbers of synaptotagmine-1 and PSD 95 induced by Aβ_1–42_ oligomers (*P* < 0.01, Figures 4M,N), while, NPS 2143 treatment alone (0.1 μM) did not alter the numbers of synaptotagmine-1-positive puncta and PSD 95-positive puncta (*P* > 0.05, Figures 4M,N). The synaptic density of the Aβ_1–42_ group was also significantly declined (*P* < 0.01, Figure 4O). The NPS 2143 (0.1 μM) treatment significantly ameliorated the reduction of synaptic density induced by Aβ_1–42_ oligomers (*P* < 0.01), while, NPS 2143 treatment alone (0.1 μM) had no effect on the synaptic density (*P* > 0.05, Figure 4O).

### Down-Regulation of CaSR Expression Prevents Synaptic Impairment Induced by Oligomeric Aβ_1–42_ in Hippocampal Neurons

**Figure 5:**
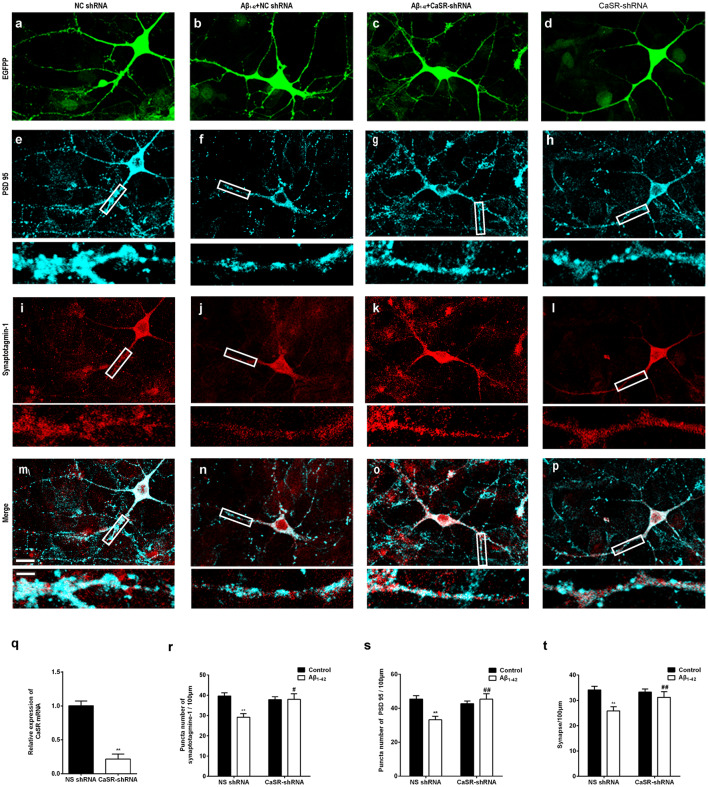
Down-regulation of CaSR expression prevented synaptic impairment induced by oligomeric Aβ_1–42_ in hippocampal neurons. Sample figures of the NC shRNA **(A,E,I,M)**, the 0.5 μM Aβ_1–42_ + NC shRNA group **(B,F,J,N)**, the 0.5 μM Aβ_1–42_ + CasR-shRNA group **(C,G,K,O)**, and the CasR-shRNA group **(D,H,L,P)**. Scale bar 20 μm and 5 μm. **(Q)** The expression level of CaSR was significantly down-regulated by CaSR-shRNA. **P < 0.01 vs. NC shRNA group. **(R,S)** Quantitative comparison of the number of synaptotagmine-1-positive puncta and PSD 95-positive puncta of the four groups. **(T)** Down-regulation of CaSR protected hippocampal neurons from synapse loss induced by oligomeric Aβ_1–42._ **P < 0.01 vs. NC shRNA group, ^#^*P* < 0.05 vs. Aβ_1–42_ + NC shRNA group, ^##^*P* < 0.01 vs. Aβ_1–42_ + NC shRNA group. Values represent mean ± SEM, *n* = 17–20 neurons from three independent primary cell culture preparations.

To further verify that CaSR mediates the synaptic impairment induced by Aβ_1–42_, the synaptic densities of neurons treated by the CaSR-shRNA-lentivirus or NC shRNA were analyzed by immunocytochemistry (Figures 5A–P). The expression level of CaSR was down regulated by the CaSR-shRNA-lentivirus (*P* < 0.01, Figure 5Q). Compared with the control group, the numbers of synaptotagmine-1-positive puncta and PSD 95-positive puncta were significantly decreased (*P* < 0.01, Figures 5R,S) in the oligomeric Aβ_1–42_-treated cells. Down-regulation of the CaSR expression significantly prevented the decreased puncta numbers of synaptotagmine-1 and PSD 95 (*P* < 0.01, Figures 5R,S). The synapse number was also measured, and down-regulation of CaSR significantly protected hippocampal neurons from synapse loss induced by oligomeric Aβ_1–42_ (*P* < 0.01, Figure 5T).

### NPS 2143 Prevents the Decreased Expression Levels of Synaptotagmine-1 and PSD 95 in AD Model Mice

Collective evidence shows that oligomeric Aβ_1–42_ is regarded as the pertinent toxic form of Aβ (Baek et al., 2017; Choi et al., 2017). In order to study the single factor of Aβ and the underlying mechanism of CaSR in Aβ-mediated synaptic and cognitive impairment, the AD mouse model, made by microinjection with Aβ_1–42_ oligomers, were used in the rest of our study.

**Figure 6:**
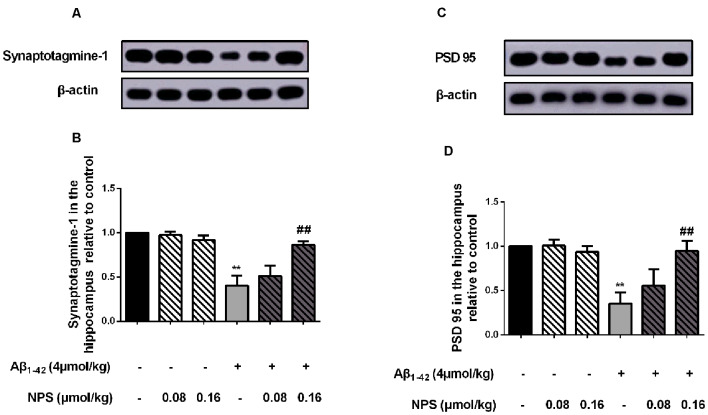
Pharmacological inhibition of CaSR prevented the decreased expression levels of synaptotagmine-1 and PSD 95 induced by oligomeric Aβ_1–42_. **(A)** Sample western-blot plot of synaptotagmine-1 and β-actin in the control, the oligomeric Aβ_1–42_-treated group (4 μmol/kg), and/or the NPS 2143-treated group (0.08 or 0.16 μmol/kg). **(B)** Quantitative comparison of hippocampal synaptotagmin-1 level in different groups. **(C)** Sample western-blot plot of PSD 95 and β-actin in six groups. **(D)** Quantitative comparison of hippocampal PSD 95 level in different groups. **P < 0.01 vs. control group, ^##^*P* < 0.01 vs. Aβ_1–42_ oligomer-treated group. Values represent mean ± SEM, *n* = 6 animals for each group.

To investigate whether CaSR also mediates the synaptic impairment in the AD mouse model, the expression levels of the presynaptic marker synaptotagmine-1 and postsynaptic mark PSD 95 were measured in the hippocampus of the mice which were microinjected with Aβ_1–42_ (4 μmol/kg) and/or (0.08 or 0.16 μmol/kg) NPS 2143 (Figures 6A,C). The expression levels of synaptotagmine-1 and PSD 95 were significantly decreased in the hippocampus of the Aβ_1–42_-treated mice (*P* < 0.01, Figure 6B; *P* < 0.01, Figure 6D). The NPS 2143 (0.16 μmol/kg) treatment significantly prevented the decreased expression levels of PSD 95 and synaptotagmine-1 (*P* < 0.01, Figure 6B; *P* < 0.01, Figure 6D).

### Pharmacological Inhibition of CaSR Prevents Cognitive Deficits of Aβ_1–42_ Oligomer-Treated Mice

**Figure 7:**
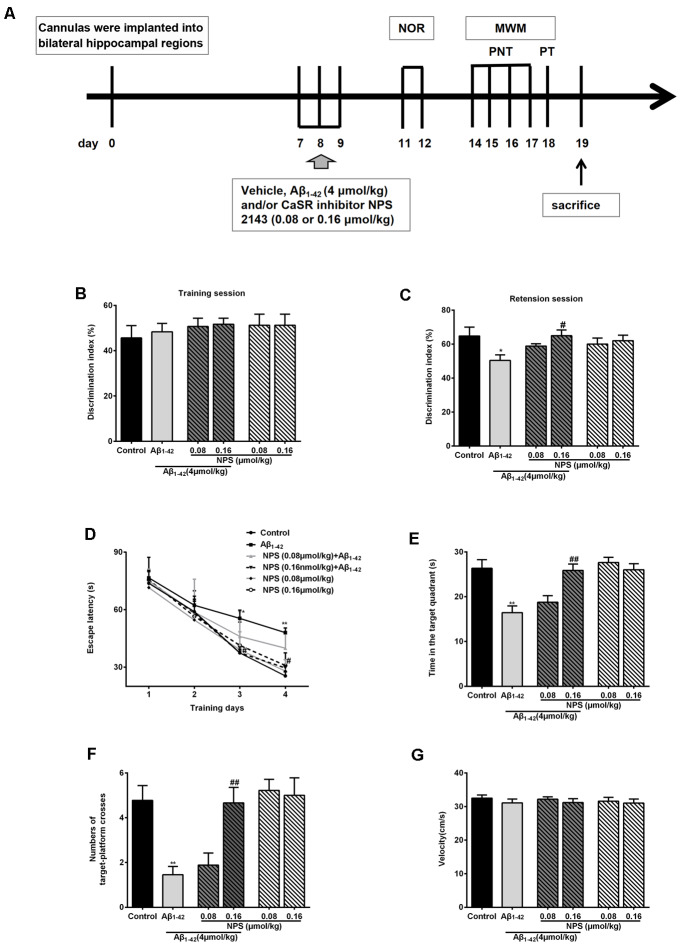
Protective function of inhibiting CaSR on oligomeric Aβ_1–42_-induced recognitive and spatial learning deficits. **(A)** Experimental schedule of behavioral tests. Cannulas were implanted into bilateral hippocampal regions. Vehicle, Aβ_1–42_ (4 μmol/kg) and/or CaSR inhibitor NPS 2143 (0.08 or 0.16 μmol/kg body weight per day) were microinjected for 3 days from day 7 to 9. From day 11 to day 12, the novel object recognition (NOR) tests were performed. Morris water maze (MWM) tests were conducted from day 14 to 18 day. Place navigation tests (PNT) of the MWM were conducted four times a day for four consecutive days, followed by a probe trial test (PT) 24 h after the last PNT. The mice were sacrificed after behavioral experiments. **(B)** Discrimination indexes displayed no significant difference among these groups in the training session. **(C)** Quantitative comparison of the discrimination indexes in the retention session, *n* = 8 animals for each group. **(D)** The escape latency of the control, the oligomeric Aβ_1–42_-treated group (4 μmol/kg), and/or the NPS 2143-treated group (0.08 or 0.16 μmol/kg) during the four training days. **(E)** Quantitative comparison of the time in the target quadrant of the six groups. **(F)** Quantitative comparison of the number of target platform crosses of the six groups. **(G)** Swimming speed of the six groups did not show significant different. *P < 0.05 vs. control group, **P < 0.01 vs. control group, ^#^*P* < 0.05 vs. Aβ_1–42_ oligomer-treated group, ^##^*P* < 0.01 vs. Aβ_1–42_ oligomer-treated group. Values represent mean ± SEM, *n* = 8 animals for each group.

Normal synapse formation is considered to be the structure basis of cognitive function. To find out whether the impairment of synapse formation mediated by CaSR was also involved in the cognitive deficits of the AD mouse model, NOR tests and MWM tests were used to estimate the role of CaSR in soluble Aβ_1–42_ oligomer induced recognitive and spatial memory deficits (Figure 7), respectively.

The discrimination indexes were used to evaluate the recognitive memory of animals in NOR tests. There was no significant difference in discrimination indexes among these groups in the training session (*P* > 0.05, Figure 7B). In the retention session, discrimination indexes were decreased in the mice injected with soluble Aβ_1–42_ oligomers (*P* < 0.05, Figure 7C). Inhibition of CaSR with NPS 2143 (0.16 μmol/kg) had no effect on discrimination indexes, but it significantly attenuated the Aβ_1–42_ oligomer-induced reduction of discrimination indexes (*P* < 0.05, Figure 7C). The NPS 2143 (0.08 μmol/kg) treatment had no effect on Aβ_1–42_-induced change in the discrimination indexes (*P* > 0.05, Figure 7C). These results indicated that NPS 2143 rescues Aβ_1–42_-induced recognitive deficits in a dose dependent manner.

To further investigate whether CaSR is involved in Aβ_1–42_ oligomer-mediated spatial memory impairment, we examined memory performance with MWM tests in mice treated with Aβ_1–42_ oligomers in presence or absence of CaSR antagonist NPS 2143. Two-way ANOVA for repeated-measures revealed significant changes in drug effects (*P* < 0.01, Figure 7D) and time effects (*P* < 0.01, Figure 7D), but no interaction was found (*P* > 0.05, Figure 7D). The escape latency of Aβ_1–42_-treated mice on day 3 and 4 was significantly longer compared with the control mice (*P* < 0.05 and *P* < 0.01 for day 3 and 4 respectively, Figure 7D). The escape latency of NPS 2143-treated mice (0.08 or 0.16 μmol/kg) was stable compared to that of the control mice. The NPS 2143 (0.08 μmol/kg) did not prevent the increase escape latency of Aβ_1–42_-treated mice, however, the NPS 2143 (0.16 μmol/kg) treatment prevented the prolongation of latency induced by Aβ_1–42_ on day 3 and 4 (*P* < 0.05, Figure 7D). In the probe test, after the hidden platform was removed from the target quadrant, the Aβ_1–42_-treated mice spent a shorter time in the target quadrant compared with the control mice (*P* < 0.01, Figure 7E). A 0.16 μmol/kg NPS 2143 treatment reversed the decrease of time in the target quadrant of the Aβ_1–42_-treated mice (*P* < 0.01, Figure 7E). However, treatment of NPS 2143 alone, at the dose of 0.08 or 0.16 μmol/kg, had no effect on the time spent in the target quadrant (Figure 7E). Moreover, we measured the numbers of times the mice swam cross the place where the original platform was. The numbers of the target platform crosses were decreased in the Aβ_1–42_-treated mice (*P* < 0.01, Figure 7F). Injection of NPS 2143 (0.16 μmol/kg) reversed the decreased crosses over the target platform of Aβ_1–42_-treated mice (*P* < 0.01, Figure 7F). However, treatment of NPS 2143 alone had no effect on the numbers of the target platform crosses (Figure 7F). We did not observe a significant difference in velocity among the six groups in the MWM tests (Figure 7G), indicating that the differences in latency, time and number of crosses over the target platform among the groups were not caused by the differences in velocity. Altogether, these data suggested that pharmacological inhibition of CaSR prevents mice from Aβ_1–42_ oligomer- induced spatial learning and memory deficits in MWM tests.

### CaSR Participates in Aβ1–42-Induced Increase in the Levels of cPLA2 and PGE2

**Figure 8:**
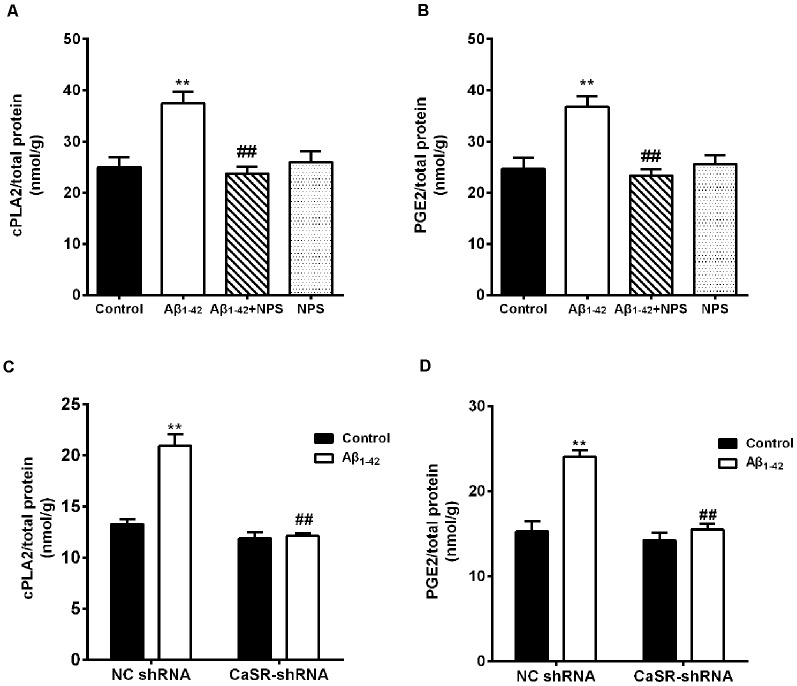
CaSR was involved in Aβ_1–42_-induced increase of cytosolic phospholipase A2 (cPLA2) and prostaglandin E2 (PGE2) in hippocampal neurons. **(A,B)** Quantitative comparison of the contents of cPLA2 and PGE2 in the Aβ_1–42_—(0.5 μM) and/or NPS 2143—(0.1 μM) treated groups. **P < 0.01 vs. control group, ^##^*P* < 0.01 vs. Aβ_1–42_ oligomer-treated group. Values represent mean ± SEM, *n* = 12–13 independent replicates. **(C,D)** Quantitative comparison of the contents of cPLA2 and PGE2 in the Aβ_1–42_ and/or CaSR-shRNA-lentivirus-treated groups. **P < 0.01 vs. NC shRNA group. ^##^*P* < 0.01 vs. Aβ_1–42_ + NC shRNA group, *n* = 9 independent replicates.

To explore the downstream pathways mediated by CaSR and soluble Aβ_1–42_ oligomers, the contents of cPLA2 and PGE2 were measured in hippocampal neurons. Aβ_1–42_ treatment increased the content of cPLA2 (*P* < 0.01, Figure 8A). NPS 2143 (0.1 μM) treatment significantly prevented the increased level of cPLA2 induced by Aβ_1–42_ oligomers (*P* < 0.01), while the level of cPLA2 remained stable under NPS 2143 (0.1 μM) alone treated neurons, compared with that of the control neurons. The expression level of CaSR was reduced by the CaSR-shRNA-lentivirus, and knocking down CaSR also prevented the increased cPLA2 content induced by Aβ_1–42_ oligomers (*P* < 0.01, Figure 8C). Aβ_1–42_ treatment also increased the content of PGE2 (*P* < 0.01, Figure 8B). The NPS 2143 (0.1 μM) treatment significantly prevented Aβ_1–42_ oligomer-induced increase of the level of PGE2 (*P* < 0.01), while, the level of PGE2 remained stable under NPS 2143 (0.1 μM) alone treated neurons, compared with that of the control neurons (*P* > 0.05 Figure 8B). Down-regulation the expression level of CaSR also prevented the increased PGE2 content induced by Aβ_1–42_ oligomers (*P* < 0.01, Figure 8D).

**Figure 9:**
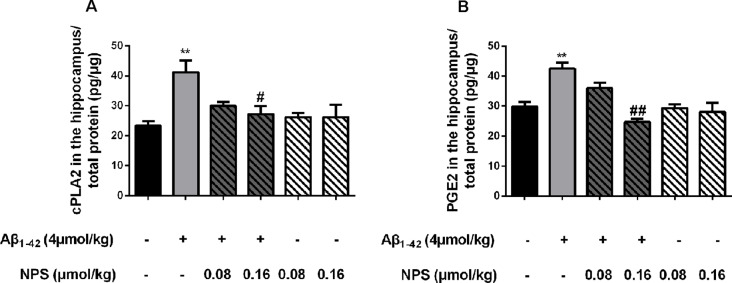
CaSR was involved in Aβ_1–42_-induced increase of cPLA2 and PGE2 in the hippocampus. **(A)** Quantitative comparison of the content of cPLA2 in the control, oligomeric Aβ_1–42_-treated group (4 μmol/kg), and/or NPS 2143-treated group (0.08 or 0.16 μmol/kg). **(B)** Quantitative comparison of the content of PGE2 in six groups. ^#^*P* < 0.05 vs. Aβ_1–42_ oligomer-treated group, **P < 0.01 vs. control group, ^##^*P* < 0.01 vs. Aβ_1–42_ oligomer-treated group. Values represent mean ± SEM, *n* = 6 animals for each group.

To verify the above results, the contents of cPLA2 and PGE2 in the hippocampus of the Aβ_1–42_ (4 μmol/kg) and/or NPS 2143 (0.08 or 0.16 μmol/kg) treated mice were also measured. The contents of PGE2 and cPLA2 were significantly increased in the hippocampus of the Aβ_1–42_-treated mice (*P* < 0.01, Figure 9A; *P* < 0.01, Figure 9B). The NPS 2143 (0.16 μmol/kg) treatment reversed the increased levels of PGE2 and cPLA2 (*P* < 0.05, Figure 9A; *P* < 0.01, Figure 9B).

## Discussion

To the best of our knowledge, our results, for the first, time demonstrate that the expression level of CaSR is increased by Aβ_1–42_. The increase expression or activity of CaSR mediates the AD-like phenotypes/pathology induced by Aβ_1–42_ oligomers partially through activation of the cPLA2/PGE2 pathway. CaSR is located in nerve terminals which are related to synaptic plasticity and neuronal transmission (Bandyopadhyay et al., 2010). In this study, we provided novel insights to the possible role and underlying mechanisms of CaSR in AD.

It is well accepted that the hippocampus is a region of the adult brain where neurogenesis occurs. Perturbations in synapse formation by forms of oligomeric Aβ are tightly correlated with memory deficits in AD (Bandyopadhyay et al., 2010; Marchetti and Marie, 2011; Ardiles et al., 2012; Ma and Klann, 2012; Sanchez et al., 2012; Xu et al., 2014). Growing evidence indicates that Aβ-induced synaptic loss in the hippocampus occurs at the early stage of AD (Teich et al., 2015; Wang X. et al., 2018). We demonstrated that exposure of Aβ_1–42_ oligomers potently decreased filopodium density in hippocampal neurons, while CaSR inhibitor NPS 2143 significantly prevented Aβ_1–42_ oligomer-induced filopodium loss. These results suggest that CaSR is involved in the negative role of Aβ_1–42_ during initial synapse formation. Dendritic spines, small membranous protrusions from neuronal dendrites, are developed from filopodia. Consistent with the role of CaSR in the Aβ_1–42_-induced decrease in filopodium density, CaSR was also involved in the impairment of spine and synapse formation mediated by Aβ_1–42_ oligomers. Both pharmacological inhibition of CaSR and knockdown of CaSR prevented Aβ_1–42_-induced synapse developmental deficits. Moreover, we found that CaSR also mediated Aβ_1–42_-induced cognitive deficits. Using behavioral tasks, including MWM and NOR tests, we also showed that Aβ_1–42_ oligomers cause recognitive and spatial memory impairment. Inhibition of CaSR with NPS 2143 prevented Aβ_1–42_-induced cognitive deficits in a dose-depended manner. Thus, CaSR mediates AD-like synaptic and cognitive impairment induced by Aβ_1–42_ oligomers.

cPLA2/PGE2 signaling pathways might be involved in the effects of CaSR on Aβ-induced cognitive impairment. PGE2, a lipid molecule derived from arachidonic acid (AA; Brummett et al., 2014). cPLA2 affects the synthesis of PGE2 by promoting the release of AA (Bate and Williams, 2015a). It has been found that the activation of cPLA2/PGE2 is associated with multiple signaling pathways such as neuronal excitation, synaptic secretion, lipid metabolism, and neuroinflammation (Murakami and Kudo, 2004; Igarashi et al., 2011; Sun et al., 2014). Activation of cPLA2 enzymes plays an important role in age-associated neuronal and memory impairment (Hermann et al., 2014). Neurons isolated from mice deficient in cPLA2^−/−^ showed resistance to the toxic effects of Aβ (Desbàne et al., 2012). PGE2 also regulates synaptic function and plasticity (Koch et al., 2010). Addition of PGE2 reduced the content of synaptic proteins in cortical neurons, and impaired hippocampal presynaptic long-term plasticity in a mouse model of AD (Bate et al., 2010; Maingret et al., 2017). Our results showed that the contents of cPLA2 and PGE2 in hippocampal neurons and hippocampal encephalic region were significantly increased due to oligomeric Aβ_1–42_ treatment. These results are consistent with previous studies showing that Aβ oligomers could activate cPLA2 and increase the level of PGE2, resulting in a reduction of synaptic markers and a decline in cognitive function (Desbàne et al., 2012; Bate and Williams, 2015b). Both pharmacological inhibition of CaSR and down-regulation of the expression of CaSR prevented Aβ-induced increase of cPLA2/PGE2 and synaptic damage. Thus, CaSR might mediate Aβ-induced synaptic and cognitive damage through increasing cPLA2 and PGE2 contents.

Besides activation of the cPLA2/PGE2 pathway, CaSR has also been reported to be involved in the Aβ-induced increase of Aβ and phospho-tau (Dal Prà et al., 2014; Chiarini et al., 2017); this might also contribute to the decrease in cognitive decline in AD mice. At physiological conditions, CaSR was only partially activated (Ruat and Traiffort, 2013; Díaz-Soto et al., 2016). Increased Aβ made CaSR full or over activated, which further induced the early impairment of synapse formation and cognitive function. Together with our observations, the current data showed that CaSR is an important factor mediating the progress of AD.

In conclusion, we demonstrated that CaSR is involved in oligomeric Aβ_1–42_-induced cognitive dysfunction as well as synapse formation and developmental impairment in the pathogenesis of AD, in addition, our results indicated that cPLA2 and PGE2 are downstream targets of CaSR, which mediate cognitive decline in AD. Thus, we provided support for the efficacy of specific antagonists of CaSR in the treatment of AD.

## Data Availability Statement

The datasets generated for this study are available on request to the corresponding author.

## Ethics Statement

The animal study was reviewed and approved by Institutional Animal Care and Use Committee of the Medical School of Ningbo University [permission: SYXK(ZHE)2013-0191].

## Author Contributions

SX and XB were responsible for the design of the study. CF was in charge of molecular and cellular experiments. YL, YD, and YC were mainly involved in animal experiments and relative analysis. QW and JW provided valuable advice for the research. WC and LS provided language modification and data analysis. All authors contributed to the article and approved the submitted version.

## Conflict of Interest

The authors declare that the research was conducted in the absence of any commercial or financial relationships that could be construed as a potential conflict of interest.

## Abbreviations

AA: arachidonic acid
Aβ: β-amyloid protein
ANOVA: analysis of variance
AD: Alzheimer’s disease
CaSR: calcium-sensing receptor
cPLA2: cytosolic phospholipase A2
DIV: day *in vitro*
F-GFP: farnesylated enhanced green fluorescent protein
GPCRs: G protein-coupled receptor
HFIP: 1,1,1,3,3,3-hexafluoro-2-propanol
LTP: long-term potentiation
MWM: morris water maze
NOR: novel object recognition
PBS: phosphate-buffered saline
PGE2: prostaglandin E2
PNT: place navigation test
PT: probe trial test
PSD 95: anti-postsynaptic density 95 antibody.
